# Adiponectin and Disease Severity in Sickle Cell Anemia Patients Attending a Tertiary Health Institution in Nnewi, Southeast Nigeria

**DOI:** 10.3389/fgene.2022.799425

**Published:** 2022-02-23

**Authors:** Chide Emmanuel Okocha, Patrick O. Manafa, Chioma Nkechinyere Igwe, Uchechukwu Prince Okite, Christian Ejike Onah, Chilota Efobi

**Affiliations:** ^1^ Haematology Department, Nnamdi Azikiwe University Teaching Hospital, Nnewi, Nigeria; ^2^ Department of Medical Laboratory Science, Faculty of Basic Medical Sciences, Nnamdi Azikiwe University, Nnewi, Nigeria; ^3^ Haematology Department, University of Port Harcourt, Port Harcourt, Nigeria

**Keywords:** adiponectin, sickle cell anemia, body mass index, sickle cell disease severity, endocrinopathy

## Abstract

**Background:** Hemoglobin polymerization in sickle cell anemia (SCA) leads to abnormally rigid and adhesive erythrocytes that obstruct blood vessels, leading to poor tissue perfusion, hence provoking inflammation and damage of surrounding tissues. Adiponectin, a protein hormone, presumptively has anti-inflammatory characteristics, hence may be an important therapeutic target in SCA.

**Aim:** The aim of the study was to evaluate the status of adiponectin and its correlation with disease severity in SCA.

**Patients and Methods:** A total of 84 subjects were recruited for the study comprising 34 homozygous sickle cell (HbSS) subjects (25 in the steady state and nine in the resolving crisis state) and 50 controls (25 heterozygous sickle cell [HbAS] and 25 hemoglobin phenotype AA subjects). The hemoglobin phenotype, adiponectin levels, and full blood counts were evaluated. Anthropometric measurements were also conducted.

**Results:** A significant difference was observed in the mean body mass index between the different hemoglobin phenotype groups and also between the SCA in crisis resolution patients and the control group (*p* < 0.05). There was no significant difference in the median serum levels of adiponectin in the different hemoglobin phenotype groups and between SCA patients in the steady state compared with those in the crisis resolution state. Also, there was no correlation between disease severity and adiponectin in SCA patients in the steady state (*p* = 0.87).

**Conclusion:** Our study seems to suggest that in our data set of sickle cell anemia patients in the steady state, adiponectin does not constitute part of the endocrinopathy that affects these patients.

## Introduction

Sickle cell disease (SCD) is a group of genetic conditions that result from the inheritance of abnormal genes, thereby resulting in the production of abnormal hemoglobin in red blood cells (Akinyanju, 2015). The most common and most severe form of SCD, sickle cell anemia (SCA), refers to homozygosity for the sickle hemoglobin (Hb), known as HbSS (Piel et al., 2017). Hemoglobin polymerization leads to abnormally rigid and adhesive red blood cells (RBCs) that obstruct blood vessels, leading to tissue damage from poor perfusion in HbSS, hence provoking inflammation, as they stimulate and damage surrounding tissues and cells (Serjeant, 1997) (Platt, 2000). Keikhaei et al. showed that pro-inflammatory cytokines, especially lL-8 and IL-17 were increased in SCD patients in the steady state when compared to those in controls (apparently normal individuals) and increased in the crisis state when compared to the steady state (Keikhaei et al., 2013). This suggests that there is a background inflammation in the steady state which escalates during crisis.

The body mass index (BMI) is broadly used to categorize a person as underweight, normal weight, overweight, or obese based on tissue mass (muscle, fat, and bone) and height (Malcolm, 2015). Historically, it is well documented that children with SCD were underweight, particularly those with HbSS due to a characteristic basal hypercatabolic metabolism (Thomas et al., 2000; Barden et al., 2000).

Adiponectin is a protein hormone that modulates a number of metabolic processes, including glucose regulation and fatty acid oxidation (Díez and Iglesias, 2003). Adiponectin is secreted from adipose tissue (and also from the placenta during pregnancy) into the blood. It is a specific protein with presumptive antiatherogenic, insulin-sensitizing, and anti-inflammatory characteristics (Chen et al., 2006; (Hotta et al., 2000; (Bełtowski et al., 2008). Adiponectin is considered important in the etiopathogenesis of many vascular and inflammatory disorders due to its anti-inflammatory actions (Zhu et al., 2008; (Cui et al., 2011). It also inhibits the synthesis of pro-inflammatory cytokines such as IL-6, IL-18, and TNF-α synthesis by blocking NF-κB activation (Yamaguchi et al., 2005; Chandrasekar et al., 2008; Ouchi and Walsh, 2017).

Exploring the role of adiponectin in sickle cell anemia hence becomes important, given that sickle cell anemia has a background chronic inflammatory state and adiponectin may be an important therapeutic target in its management.

## Subjects and Methods

### Patient Selection

A total of 84 subjects were recruited for the study from the hematology clinic of a tertiary health center and during community interactive sessions with patients. These included 25 homozygous sickle cell (HbSS) subjects in the steady state [the selection of the steady state group was dependent on subjects’ not experiencing crisis for at least 4 weeks, not receiving blood transfusion for at least 3 months, and not having fever for at least 2 weeks prior to the study (Okocha et al., 2014)]. A total of nine homozygous sickle cell (HbSS) patients, whose crisis was resolving, were defined as HbSS patients who did not meet the criteria for the steady state disease but were not in the acute phase of SCA crisis; while 25 heterozygous sickle cell (HbAS) and 25 hemoglobin phenotype AA subjects were used as controls. The ethical approval for this research was obtained from the Nnamdi Azikiwe University Teaching Hospital Ethics Committee, and written informed consent was sought and received from the subjects or their caregivers.

### Disease Severity Score in Hemoglobin SS Patients

Disease severity was calculated using an objective severity scoring system, where points were assigned using the following characteristics: number of hospital admissions for crisis per year, rate of transfusions, white blood cell count, number of complications, and the degree of anemia. Scores of ≤3 were considered mild disease; scores of >3–≤ 7, moderate disease; while scores of >7 were considered severe disease (Okocha et al., 2017).

### Specimen Collection, Preparation, and Storage

About 5 ml of venous blood was collected aseptically by venipuncture from each subject *via* the antecubital vein using a plastic syringe with minimum stasis, and 2 ml was then dispensed into ethylene diamine tetra acetic acid bottles for the determination of the hemoglobin phenotype and the full blood count. The remaining 3 ml was emptied into a plain bottle and centrifuged at 4000 rpm for 10 min. The serum (supernatant) was stored at −22°C and then used for the determination of adiponectin levels. The hemoglobin phenotype was determined using the Zip Zone electrophoresis chamber and EV 243 power supply (Helena Biosciences, United Kingdom). The adiponectin level was determined using commercially available adiponectin test-kits, and its assay was based on enzyme-linked immunosorbent assay. The full blood count was performed using a Sysmex automated hematology analyzer (KX2IN model, Sysmex Corporation, Kobe, Japan)

### Statistical Analysis

Data obtained were analyzed using the Statistical Package for Social Sciences software package version 20 (SPSS Inc., IL, Chicago, United States). Descriptive statistics were used to summarize the variables and characterize the demographics. The statistical analysis was performed using the Kruskal–Wallis test to compare the differences in medians between two and three groups. The Spearman’s correlation coefficient was used for correlation of nonparametric variables. The values were deemed significant when *p* < 0.05.

## Results

There was a total of 84 subjects recruited for this study which included 25 homozygous sickle cell (HbSS) subjects in the steady state, nine homozygous sickle cell (HbSS) patients whose crisis were resolving, 25 heterozygous sickle cell (HbAS) subjects, and 25 hemoglobin phenotype AA subjects. The HbSS subjects in the steady state were 14 nonpregnant females and 11 males; HbSS in crisis resolution included eight females and one male; HbAS included 16 males and 9 females, while HbAA included 14 males and 11 females. Their age range was between 10 and 48 years with the median age of the homozygous sickle cell subjects in the steady state being 25.50 years; the median age for the homozygous sickle cell subjects in crisis resolution was 27.00 years and that for the heterozygous sickle cell subjects was 23.00 years and the HbAA controls was 24.00 years (Table 1). Table 1 also shows the mean BMI (kg/m) in the various hemoglobin phenotype groups. A significant difference was observed in the mean BMI between the different hemoglobin phenotype groups. However, there was no significant difference in the mean BMI between the homozygous sickle cell anemia patients in the steady and crisis resolution states and between the sickle cell anemia in crisis resolution and the control group (*p* > 0.05).

**TABLE 1:** Anthropometric values in different hemoglobin phenotype groups.

Group		N	Age (year)	BMI (kg/m^2^)
HbSS genotype	Steady (A)	30	25.50	18.58
	Crisis (B)	10	27.00	18.95
AS (C)		25	23.00	23.00
AA (D)		25	24.00	21.52
Kruskal–Wallis H			2.261	29.363
*p*-value			0.520	<0.001
A vs. B (*p*-value)			1.000	1.000
A vs. C (*p*-value)			1.000	0.006
A vs. D (*p*-value)			1.000	0.004
B vs. C (*p*-value)			1.000	0.139
B vs. D (*p*-value)			1.000	0.113
C vs. D (*p*-value)			1.000	1.000

Table 2 shows the median levels of adiponectin in the different hemoglobin phenotype groups. There was no significant difference in the median serum levels of adiponectin in the different hemoglobin phenotype groups and between sickle cell anemia patients in the steady state and crisis resolution state.

**TABLE 2:** Levels of Adiponectin in different hemoglobin phenotype groups.

Group		N	Adiponectin (µg/L)
HbSS genotype	Steady (A)	25	34.00
	Crisis resolution (B)	9	37.00
AS (C)		25	33.50
AA (D)		25	34.00
Kruskal–Wallis H			0.579
*p*-value			0.878
A vs. B (*p*-value)			1.000
A vs. C (*p*-value)			1.000
A vs. D (*p*-value)			1.000
B vs. C (*p*-value)			1.000
B vs. D (*p*-value)			1.000
C vs. D (*p*-value)			1.000

**FIGURE 1:**
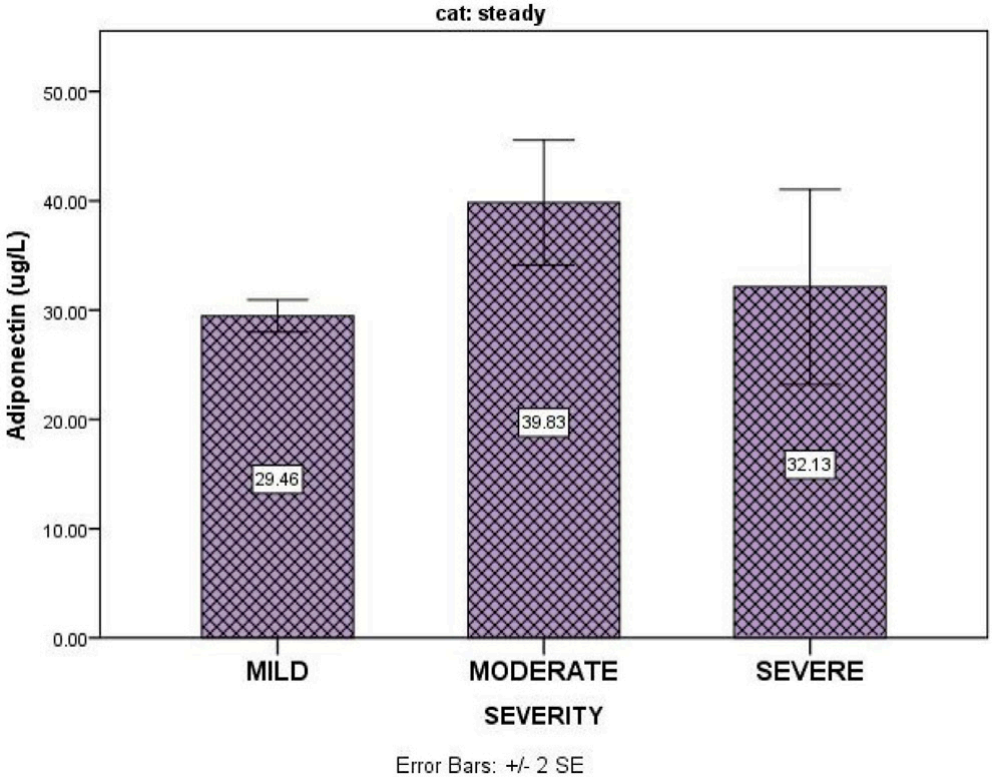
SCD severity in the steady state versus adiponectin levels.

Table 3 shows that disease severity in SCA patients in the steady state had no correlation with serum adiponectin (*p* = 0.87). Figure 1 shows the relationship between serum levels of adiponectin and sickle cell disease severity for subjects in the steady state.

**TABLE 3:** Correlation studies of BMI, age, and adiponectin levels with disease severity in subjects with sickle cell anemia in the steady state.

Parameter	N	R	*p*
Disease severity vs. BMI	30	−0.012	0.948
Disease severity vs. age	30	−0.035	0.853
Disease severity vs. adiponectin	25	0.032	0.870

## Discussion

We have shown from this study that the mean serum adiponectin levels were not significantly different amongst the subjects from the different hemoglobin phenotypes, which is in contrast to the findings by Makis et al., who reported elevated adiponectin levels in steady state sickle cell anemia patients when compared with control subjects. This seems to suggest that in our data set of sickle cell anemia patients in the steady state, adiponectin does not constitute part of the endocrinopathy that affects these patients. Genetic variation could be the likely explanation of this difference between our data set and that of Makis et al. (2012).

The finding from this study reveals that the BMI is significantly decreased in sickle cell anemia in the steady state when compared with that of the control group (HbAA) is corroborated by Odetunde et al. (2016). Poor growth and nutrition are common in children with sickle cell anemia (SCA), which was demonstrated by Oredugba et al. in which the nutritional status in children with SCA negatively affected the anthropometric status, disease severity, and body composition of the patients (Oredugba, 2002). Some have hypothesized that underweight in SCA is caused by a hypermetabolic state, which increases energy demand that may lead to an undernourished state if not offset by increased nutrient consumption (Hibbert et al., 2006). Increased expenditure of energy at rest is a major metabolic change associated with HbSS. Barden et al. reported that energy expenditure at rest is approximately 15–20% higher in adolescents with HbSS than HbAA (Barden et al., 2000). This study showed no significant disparity in the BMI of subjects in the steady state when compared to those in crisis resolution, which is in agreement with the study by Iwalokun et al. (2011). This may be because the crisis duration may not be long enough to significantly alter the patients weight.

Although Makis et al. found a positive correlation between adiponectin and inflammatory markers, we found no correlation between adiponectin and disease severity. This may be because our disease severity scoring system is a composite of many parameters and therefore is more robust in predicting actual disease severity.

Our finding agrees with the report by Hall et al. (2018) which showed no association between BMI of sickle cell anemia subjects and disease severity. Also, no correlation was found between age and disease severity, which contrasts with the study by Adegoke and Kuti (2013), who found a positive correlation between age and disease severity.

## Conclusion

Our data suggest that adiponectin does not seem to constitute part of sickle cell endocrinopathy in steady state sickle cell anemia patients at Nnewi, Southeast Nigeria. Also from the study, we found that the subjects with sickle cell anemia in crisis resolution and steady states had lower BMI values than HbAA and HbAS subjects. We therefore recommend that the nutritional needs of patients living with sickle cell anemia should be given more attention to improve the general well-being of the patients.

### Limitation of the Study

Although this study has presented some insight into the dynamics of adiponectin in SCA clearly, it is limited because a larger longitudinal study starting from when the patients are in steady state through crisis state would have given further insight into the dynamics of adiponectin and BMI in these patients, thereby making for a better patient treatment, monitoring, and outcome.

## Data Availability

The original contributions presented in the study are included in the article/Supplementary Material; further inquiries can be directed to the corresponding author.
